# Comparing design students’ inter-brain synchrony between face-to-face and online collaborations

**DOI:** 10.1007/s00429-026-03188-4

**Published:** 2026-09-04

**Authors:** Luqian Wang, Clive HY Wong, Kin Wai Michael Siu, Bolton KH Chau, Zhen Yuan, Leanne Chang, Yi-Teng Shih

**Affiliations:** 1 School of Design, The Hong Kong Polytechnic University, Hong Kong, China; 2 Department of Psychology, The Education University of Hong Kong, Hong Kong, China; 3 Department of Rehabilitation Sciences, The Hong Kong Polytechnic University, Hong Kong, China; 4 Centre for Cognitive and Brain Sciences, University of Macau, Macau, China; 5 Department of Communication Studies, Hong Kong Baptist University, Hong Kong, China

**Keywords:** Inter-brain synchrony (IBS), Hyperscanning, Functional near-infrared spectroscopy (fNIRS), Face-to-face collaboration, Online collaboration

## Abstract

The rapid digitalisation of design education has fundamentally altered how students collaborate; however, the cognitive and neurophysiological dynamics underlying online creative co-creation remain poorly understood. To address this, we conducted an exploratory pilot study (*N* = 11 dyads) employing a multimodal paradigm that integrated behavioural protocol analysis with fNIRS hyperscanning. Specifically, this study examined the effects of collaborative modality (face-to-face versus online via Zoom) and interactional behaviours between design students on neural coupling, utilising Phase Locking Value (PLV) to quantify inter-brain synchrony (IBS). The results revealed an inversion between observable behaviour and underlying neural synchrony. In face-to-face settings, dyads spent the majority of their time communicating verbally, yet their highest levels of IBS were associated with sketching. Conversely, although participants sketched significantly more often in the online environment, the neural coupling previously associated with sketching collapsed. Instead, verbal communication emerged as the primary correlate of enhanced IBS in the digital space. These initial findings indicate that online collaborative dyads may rely more heavily on explicit verbal channels to maintain cognitive alignment. Crucially, because generative “Solution Space” activities inherently demand the visual externalisation of ideas, this disruption of sketching-associated neural coupling raises the theoretical hypothesis that remote environments may leave students more susceptible to misaligned mental models or design fixation during the solution exploration phases. It also highlights a critical avenue for future outcome-based research.

**Supplementary Information:**

The online version contains supplementary material available at https://doi.org/10.1007/s00429-026-03188-4.

## Introduction

Driven by globalisation and the imperative for digital transformation, distance collaboration has rapidly evolved into a dominant paradigm across educational and organisational spheres, enabling access to global talent and cross-disciplinary knowledge. Online collaboration typically enables geographically dispersed individuals to address a shared problem through digital tools such as video conferencing, real-time messaging and shared workspaces (Wang 2010). In the creative industries, as design thinking is mobilised to address wicked problems, the designer’s role has fundamentally expanded from a solitary innovator to a “co-creator” who requires the orchestration of interdisciplinary and cross-cultural teams (Dorst 2019; Wilson and Zamberlan 2017). Consequently, navigating these digitally mediated environments has become an essential competency for design students. Investigating how to design and implement digital tools that effectively scaffold online creative collaboration, as well as how to optimise students’ collaborative learning efficacy, has thus become a central pillar of contemporary educational technology research (Bach and Thiel 2024; Oyarzun et al. 2025).

Design education has predominantly adopted a studio-based collaborative approach, where students work in pairs or small groups to address problems, complete tasks, or produce artefacts, promoting active rather than passive learning (Joseph-Mathews et al. 2022). Rooted in Vygotsky’s (1978) sociocultural theory and Lave and Wenger’s (1991) situated learning paradigm, it rejects the view of learning as an isolated cognitive act, emphasising that knowledge is socially mediated and contextually rooted. The effectiveness of collaborative learning aligns with the design process, which Donald Schön (1983) describes as a “reflective conversation with the situation,” emphasising subjective experience, tacit knowledge, and interaction. Designers operate amid uncertainty, framing problems through engagement with materials and stakeholders, making design problem-solving an iterative process of exploring both problem and solution spaces (Maher and Poon 1996). This contrasts with rational problem-solving models (Hayes 2016; Simon and Hayes 1976).

Successful collaboration depends on the coordination of cognitive and social processes, including shared understanding, perspective-taking, and knowledge development (Johnson et al. 2007). However, Scager et al. (2016) argue that such high-quality group interactions are difficult to achieve in practice, as learners often engage only superficially. Transitioning these processes online exacerbates these challenges and introduces new hurdles. Online collaboration tends to require more explicit coordination and task management, as opposed to the implicit social cues present in face-to-face settings (Vuopala et al. 2015). Ran et al. (2025) found that online collaboration incurs a higher cognitive cost to achieve comparable results to in-person work, due to the lack of visual cues that hinder information exchange and social interaction. Lin et al. (2023) further report that remote research teams are less likely to produce innovative discoveries, as online collaboration often defaults to late-stage technical execution rather than early-stage conceptual integration. Ye et al. (2021) emphasise the importance of visual objects, such as sketches and prototypes, for conveying design ideas. Their study noted a decrease in co-exploration during remote prototyping, since the absence of shared physical interaction disrupts visual reasoning, which is the core process of generating and analysing ideas with visual tools (Goldschmidt 1991). While these studies offer valuable insights, many rely on self-reports and subjective observations.

Recent advances in “second-person neuroscience” offer a promising, objective alternative by measuring inter-brain synchrony (IBS) (Dumas et al. 2010; Czeszumski et al. 2020). IBS denotes the temporal alignment of neural oscillations between interacting individuals. This phenomenon is increasingly recognised as a neurobiological indicator of team effectiveness, empathy, joint attention and social facilitation (Du et al. 2022; Gvirts and Perlmutter 2019; Reinero et al. 2021). A recent meta-analysis of 41 hyperscanning studies involving 1326 teams found statistically significant positive associations between inter-brain synchrony (IBS) and teamwork performance (Réveillé et al. 2024). In educational contexts, Dikker et al. (2017) demonstrated that brain-to-brain synchrony predicted student engagement and social dynamics during regular classroom activities, suggesting that synchrony is driven by shared attention mechanisms. Similarly, Azhari et al. (2025) examined the online learning environment and found that higher IBS emerged during active discussion sessions compared with passive lecture listening, indicating that social involvement arouses neural alignment.

Several studies have examined how different communication modes influence neural coupling. Importantly, since inter-brain synchrony depends heavily on the task, the effects of these modalities vary with the specific collaborative context. For example, Jiang et al. (2012) found that face-to-face interactions increased neural synchrony in the left inferior frontal cortex compared with back-to-back communication during unstructured dialogue, emphasising the role of eye contact and shared physical space. Liu et al. (2019) reported higher interpersonal neural synchronization in the prefrontal cortex (PFC) during face-to-face teacher-student instruction than during computer-mediated instruction. In creative tasks such as problem-solving, product design, or alternative uses, studies consistently show increased IBS in the PFC and right temporoparietal junction (rTPJ) (Liang et al. 2022; Mayseless et al. 2019). However, results regarding digital media and these creative activities are mixed. Cheng et al. (2026) found that video-based communication disrupted the neural coupling balance typical in physical settings, whereas some studies suggest online collaboration can achieve similar IBS if adequate visual cues are provided, though it requires more cognitive effort* (*Ran et al. 2025; Wikström et al. 2022).

Despite these foundational insights, a critical research gap remains. Very few neuroscientific studies of creative collaboration examine the complex, moment-to-moment behavioural dynamics that constitute design co-creation. It remains unknown how distinct interactional behaviours for idea externalisation moderate the influence of collaborative modality (face-to-face versus online/digitally mediated) on neural coordination. This study investigates differences in interaction behaviour and neural coordination among design students across face-to-face and online synchronous collaboration modes. By bridging behavioural protocol analysis and hyperscanning, we seek to provide a holistic understanding of how digital environments affect group dynamics. We posit that IBS offers a uniquely objective lens on the “black box” of design co-creation. By correlating neural synchrony with behavioural patterns, this research responds to the field’s call for greater methodological rigour and evidence synthesis. We aim to address the research question: How do distinct interaction behaviours and the cognitive search dimension (problem- and solution-search spaces) affect student pairs’ IBS when collaboratively addressing a shared design problem?

## Literature review

### Design co-exploration

**Fig. 1:**
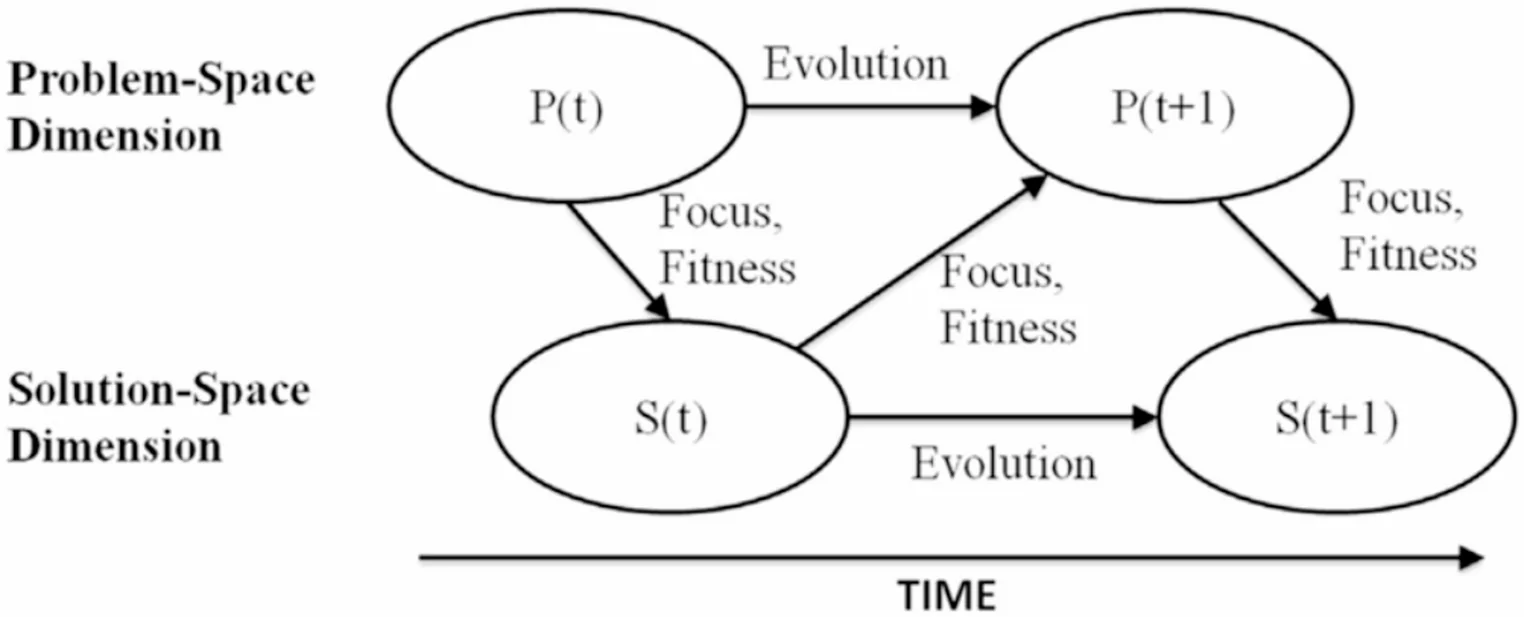
The design co-evolution model (Maher 1996)

Designing is inherently a dynamic, exploratory process, distinct from linear problem-solving. Because design problems are often open-ended challenges, neither “consistent nor complete”, they must be reframed and reinterpreted as new ideas and constraints emerge (Dorst and Cross 2001). This core dynamic is captured by the model of design co-evolution (see Fig. 1), originally conceptualised in (Maher’s work 1996) and subsequently established as a foundational cognitive model in design research (Cash et al. 2023; Dorst and Cross 2001; Maher and Tang 2003). The model posits an iterative process in which solution alternatives are explored and evaluated against problem requirements; the problem itself is simultaneously reformulated by the variables introduced through solution attempts. This reciprocal interplay aims to construct a fitting problem-solution pair, effectively proposing a “good enough” solution that satisfies a coherent set of design requirements.

In a collaborative context, this cognitive mechanism expands into design co-exploration. Co-exploration is a collaborative practice in which two or more individuals work in concert to investigate, negotiate, and navigate a shared design task. Crucially, it must be distinguished from mere coordination or cooperation. While cooperation involves dividing tasks towards a common goal (Kvan 2000), co-exploration implies a state of interdependency in which partners synchronise their cognitive trajectories in real time. In this state, the ‘problem-solution’ iteration is no longer an individual cognitive loop but a shared, distributed process. In this environment, individual cognitive processes become deeply interconnected; the contributions of one actor reciprocally shape the cognitive landscape in which their partners’ ideas develop (Sauder and Jin 2016). Empirical work by Wiltschnig et al. (2013) underscores this dynamic, capturing 63 naturally occurring co-evolution episodes in real-world design teams and observing the significance of these segments in moving design activities forward and catalysing collaborative creativity.

Building on this, a recent study by Ye et al. (2021) provides a detailed characterization of co-exploration, challenging the view that it only occurs during the divergent ideation phase. They argue co-exploration happens throughout the design process and creates “collective intelligence” that exceeds individual contributions by enabling team members to build on ideas and update shared mental models (Badke-Schaub et al. 2007). While observational studies reveal what occurs during collaboration, a gap remains in understanding the biological mechanisms enabling multiple brains to ‘synchronize’ problem-solving strategies in real-time. A neuro-oriented approach could help explore these mechanisms, moving beyond behavior to understand what drives synchronization and why it sometimes fails.

### The role of inter-brain synchrony (IBS) in design co-exploration

Inter-brain synchrony (IBS) is derived from hyperscanning techniques that measure the neurobiological coupling that occurs during naturalistic social behaviours. This synchrony is increasingly recognised as a distinct neural signature of successful social coordination, tracking phenomena ranging from simple oral communication to complex problem-solving.

Design practices are inherently ill-structured and rely on abductive reasoning to frame problems and synthesise solutions simultaneously. Scholars have observed that the neural mechanisms underlying design problem-solving differ significantly from those of linear problem-solving (Auernhammer et al. 2021). For example, neuroimaging studies demonstrate that navigating open-ended design tasks recruits a more extensive neural network and divergent neural states, particularly within the prefrontal cortex, to manage the cognitive conflict of defining ambiguous evaluation criteria, a demand does not present in well-structured tasks* (*Liu et al. 2018; Alexiou et al. 2011).

A substantial body of research suggests that the prefrontal cortex (PFC) functions as a central hub during design ideation, coordinating a wide range of cognitive processes. These include maintaining complex constraints in working memory, demonstrating cognitive flexibility to transition between alternative solutions, and exerting executive control to generate novel outputs* (*Milovanovic et al. 2021). Specifically, the right PFC plays a critical role in divergent thinking, with increased synchronisation within this region correlating with greater solution originality (Geol et al. 1997). A key sub-region, the right dorsolateral prefrontal cortex (r-DLPFC), is particularly engaged when addressing ill-structured, open-ended design challenges that necessitate the formulation of alternative hypotheses and exploration of the problem space (Gilbert et al. 2010). Conversely, the left PFC, which includes the superior, middle, and inferior frontal gyri, is consistently associated with the top-down, analytical aspects of design ideation (Campbell et al. 2024). These regions from the executive control network (ECN), underpin cognitive functions essential for idea evaluation, decision-making, logical reasoning, and the decomposition of complex problems into manageable sub-problems. Consequently, measuring inter-brain synchronisation (IBS) within the PFC offers a direct and highly pertinent insight into the collective executive efforts underpinning successful design co-creation.

While inter-brain synchrony has traditionally been studied in physical co-presence, recent evidence confirms that robust neural synchronization can emerge even among physically isolated individuals collaborating in purely digital environments, driven entirely by shared attention and joint intentionality (Wikström et al. 2022). Historically, hyperscanning literature has generally found that face-to-face (F2F) interaction facilitates higher overall neural synchrony due to the seamless integration of rich non-verbal cues and shared physical space. However, several studies reveal that digital modalities can sometimes elicit higher or more rigidly sustained levels of inter-brain synchrony within specific executive and mentalizing networks, yet yield inferior creative performance compared to physical co-presence (Wu et al. 2025; Cheng et al. 2026). A recent fNIRS-hyperscanning study elucidates that this elevated synchrony in remote settings reflects a “compensatory cognitive effort” (Wu et al. 2025). Because remote and video-mediated communication lack rich non-verbal cues, designers are forced to heavily recruit executive control (rDLPFC) and mentalizing (rTPJ) networks to maintain understanding and monitor partners. Crucially, dynamic network analyses reveal this strain forces online dyads into a rigid “high-integration” neural state (Cheng et al. 2026). This reliance on explicit cognitive alignment creates an integration bias that suppresses “neural decoupling”, a low-friction, independent cognitive state needed for divergent ideation. In physical settings, the richness of the medium allows designers to oscillate between shared cognitive alignment and neural decoupling, maximizing collaborative flexibility.

Existing hyperscanning research often treats collaborative modality as a uniform condition, reporting overall changes in inter-brain synchrony without examining the specific interactional behaviours that drive these neural shifts. A systematic review (Cheng et al. 2024) highlights that inter-brain synchrony (IBS) is particularly strong in tasks requiring precise temporal coordination, such as joint music-making. This aligns with Lübbert et al. (2021), who suggest that shared rhythms in conversation, music, or problem-solving facilitate neural coupling. These studies emphasise that IBS is dynamic, fluctuating with task interactivity. Comparisons of face-to-face and online modalities have mainly focused on verbal or text-based tasks, overlooking a key aspect of design cognition: sketching. In generative design, sketching is crucial for offloading visuospatial working memory, serving as an external memory that allows manipulation of visual features without taxing internal cognitive resources. This study aims to explore how collaborative modality (face-to-face versus online) affects inter-brain synchrony during a creative design task, using hyperscanning and behavioural analysis to examine how interactional behaviours influence neural coordination.

## Methodology

### Participant

The pilot study involved 11 pairs (N = 22, 8 females and 14 males) of final-year product design students from Hong Kong (M = 21.70, SD = 1.25). To encourage active communication and reduce social inhibition, participants were intentionally paired with individuals they already acquainted with. All were right-handed, in good health, and reported no history of neurological or visual impairments. All participants provided written consent prior to their participation. The research protocol was reviewed and approved by the Institutional Review Board of The Hong Kong Polytechnic University.

### Experimental implementation

**Fig. 2:**
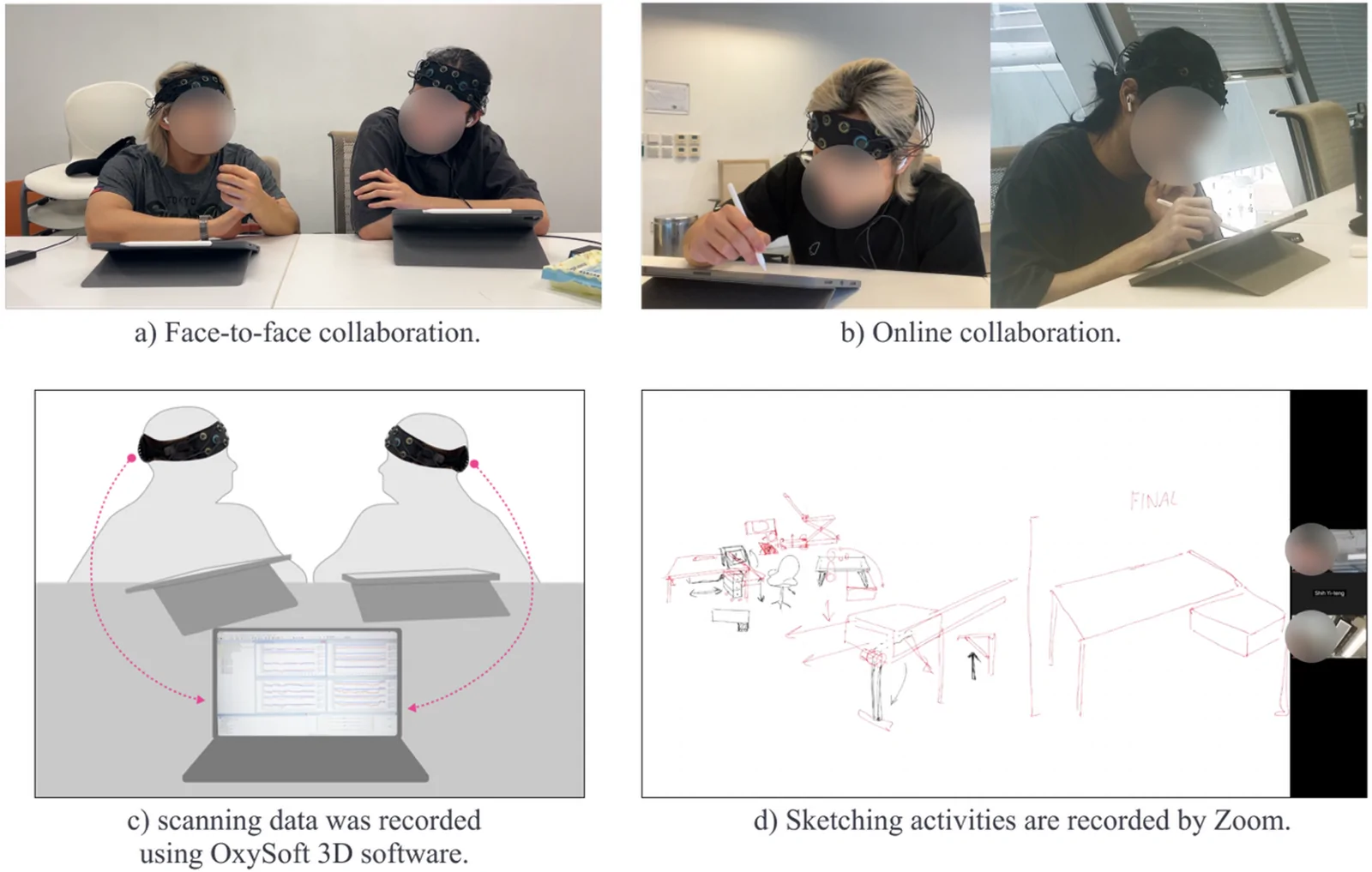
Experimental implementation

The study was conducted in a loosely controlled environment (Zhao et al. 2020) designed to support naturalistic collaboration. Each dyad wore functional near-infrared spectroscopy (fNIRS) headcaps to enable simultaneous hyperscanning of prefrontal cortex activity (see Fig. 2). Pairs were randomly assigned one of three generative design prompts for each condition. Although covering distinct themes, all prompts were explicitly designed to be ill-structured, required equivalent levels of visuospatial reasoning, and were situated in accessible, everyday contexts to eliminate the need for specialised technical expertise. The three prompts were: (1) Designing an interactive toy product to foster collaborative play and social skills among children; (2) Designing versatile, space-saving furniture adaptable for micro-living spaces; (3) Designing an intervention to mitigate and manage food waste within a campus dining environment. During the collaboration session, participants jointly sketched on a shared whiteboard on Zoom using an iPad (see Fig. 2-a). To ensure comprehensive data collection, the entire session was recorded using two methods: a dedicated camera captured non-verbal cues and physical interactions, while Zoom’s video recording function captured the on-screen sketching activities and all utterances.

**Fig. 3:**
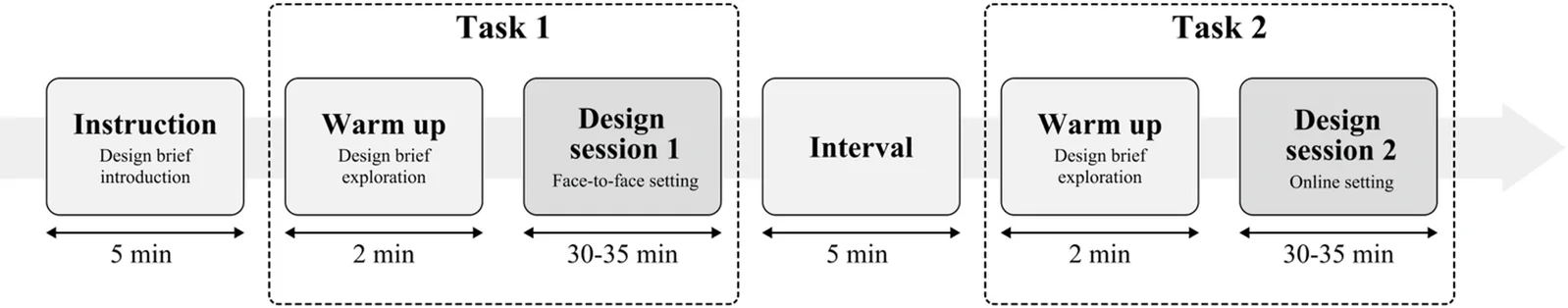
Experimental procedure. The actual chronological order of Task 1 and Task 2 was counterbalanced across dyads to prevent order effects

The experimental procedure was structured to ensure consistency across all sessions (see Fig. 3). It commenced with an instructional session introducing the design briefs. The study employed a within-subjects design comprising two primary phases: Design task 1 was conducted face-to-face, while Design task 2 occurred in the video-mediated communication environment. Both tasks began with a two-minute warm-up for brief exploration, followed by a collaborative design session lasting approximately 30 to 35 min. To systematically control for potential order, cognitive fatigue, and practice effects, the chronological sequence of the two sessions was strictly counterbalanced across the dyads (i.e., five dyads completed the face-to-face condition first, while the rest completed the online condition first). Participants were given the autonomy to conclude each session based on their subjective design progress. A five-minute interval served as a transition between the two experimental conditions. Crucially, to control for the affordances of the digital interface, participants used the identical shared iPad Zoom whiteboard interface for sketching in both the face-to-face and digital conditions. Thus, the primary manipulated variable was the dyad’s physical co-presence rather than the sketching medium itself.

### Protocol analysis

To systematically examine the cognitive underpinnings of synchronous collaborative design, this study employs protocol analysis. It is a well-established research methodology used to elicit and analyse the sequence of thoughts during a task. By capturing and segmenting participants’ verbal and non-verbal interactions, protocol analysis serves as a “window” into the distribution of cognition and the problem-solving strategies employed in both face-to-face and online settings. It transforms raw qualitative data into a structured quantitative format, enabling rigorous mapping of design behaviours to specific cognitive categories.

Two independent coding schemes were developed to segment the protocol data for a multidimensional view of the collaborative process. “Design Space Focus” (see Table 1) categorises the conceptual trajectory, distinguishing problem-sensing (students negotiate constraints and define requirements) from solution-generation (students raise and synthesise ideas). “Interaction Behaviour” (see Table 2) was defined to categorise the diverse modes, from verbal assertions to graphical representations, capturing how design intent is shared and modified within a dyad. These schemes enable detailed analysis of how communication modes (face-to-face vs. online) influence design focus and behavioural methods in understanding and knowledge co-construction.

To establish reliability, two well-trained coders, each with over three years of design education, independently apply the finalised coding schemes to a subset of the segmented data. The level of agreement between the coders will be calculated using Cohen’s Kappa (McHugh 2012), a statistical measure of inter-rater reliability. Any disagreements must be discussed to refine the operational definitions of the episodes until a high level of agreement (*κ = 0.82*) is consistently achieved.

**Table 1:** Coding scheme 1 - Design space focus

Code	Category name	Operational definition	Example utterance
PS	Problem space	1) Clarifying stakeholders, target audiences and their requirements within specific contexts; 2) Clarifying design objectives - the purpose of their design outputs (product, system, interface etc.); 3) Interpreting or shifting a previously defined requirement; 4) Defining a novel design requirement; 5) Discarding an existing requirement.	“**B**: We need a scenario to teach children something, so we need to figure out what skills are necessary, for example, cognitive skills, emotional control. **A**: Right, children tend to be quite self-centred. **B**: Yes, so they need to learn to listen to others, and they also need some patience. **A**: And self-confidence too.”
SS	Solution space	1) Proposing ideas aiming to solve the design problems they framed; 2) Modifying and improving the prior proposed ideas; 3) Evaluating design solutions from various perspectives, such as usability, material, manufacture, packaging, promotion etc.; 4) Rejecting the existing idea alternatives.	**“A**: In this way, everyone can provide a set of blocks with a board in the middle and reorganize the blocks according to each other. The blocks can have patterns inside, and the children can exchange blocks with each other based on the information provided. **B**: Will the shape be better? **A**: The shape may be too complicated.”

### fNIRS data acquisition and analysis

**Fig. 4:**
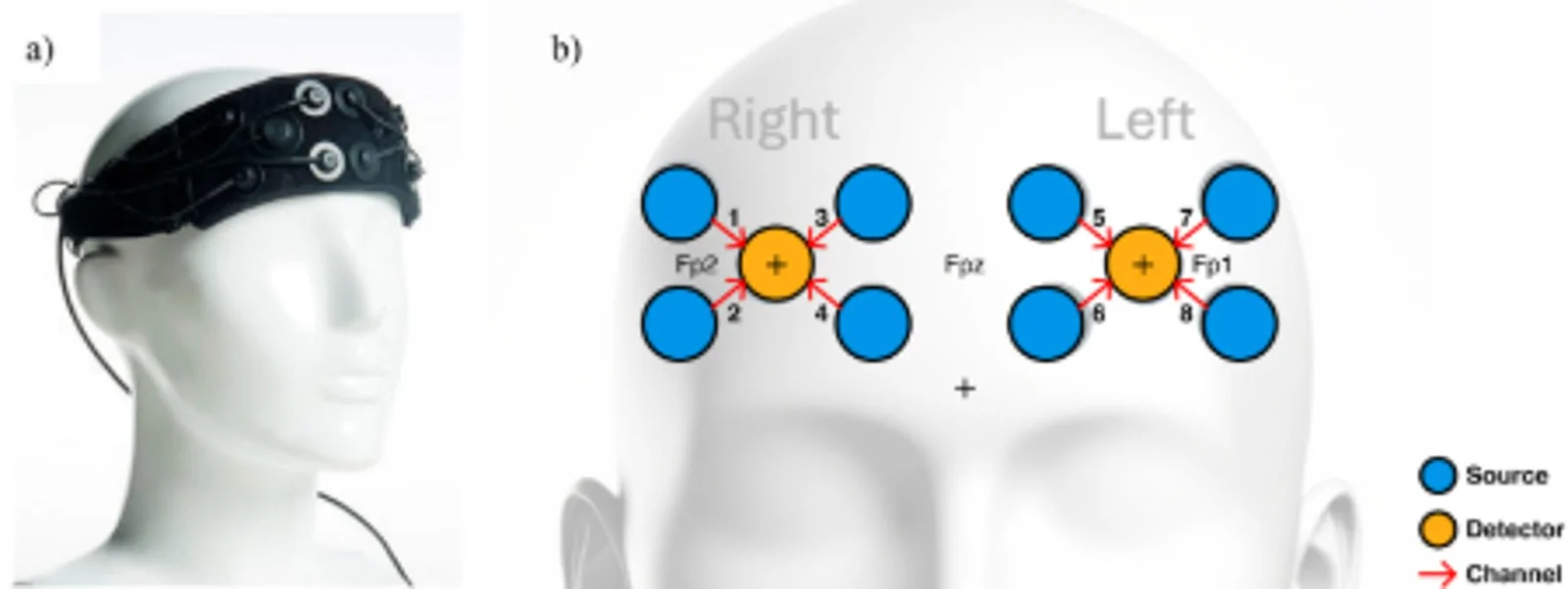
**a** The Artinis OctaMon fNIRS headband. **b** Spatial configuration of the 8 channels over the prefrontal cortex. The two receivers (orange nodes) were anchored to the international 10–20 system landmarks Fp2 (Right) and Fp1 (Left), aligning the centre of the headband with FPz.

Participants’ prefrontal cortex (PFC) activity was measured using an eight-channel functional near-infrared spectroscopy (fNIRS) system from Artinis Medical Systems, Netherlands. As shown in Fig. 4, optodes were positioned on the forehead according to the international 10/20 system to record changes in oxygenated (HbO) and deoxygenated (HbR) haemoglobin at a sampling rate of 10 Hz. The raw optical density signals were converted using the modified Beer-Lambert law (Matcher and Cooper 1994), with extinction coefficients ε(HbO₂, 850 nm) = 348.6 cm⁻¹M⁻¹, ε(HbR, 850 nm) = 569.4 cm⁻¹M⁻¹, ε(HbO₂, 760 nm) = 662.0 cm⁻¹M⁻¹, ε(HbR, 760 nm) =1355.0 cm⁻¹M⁻¹, a differential pathlength factor of 6.0 (Scholkmann and Wolf 2013), and a source-detector separation of 3.0 cm.

**Table 2:**
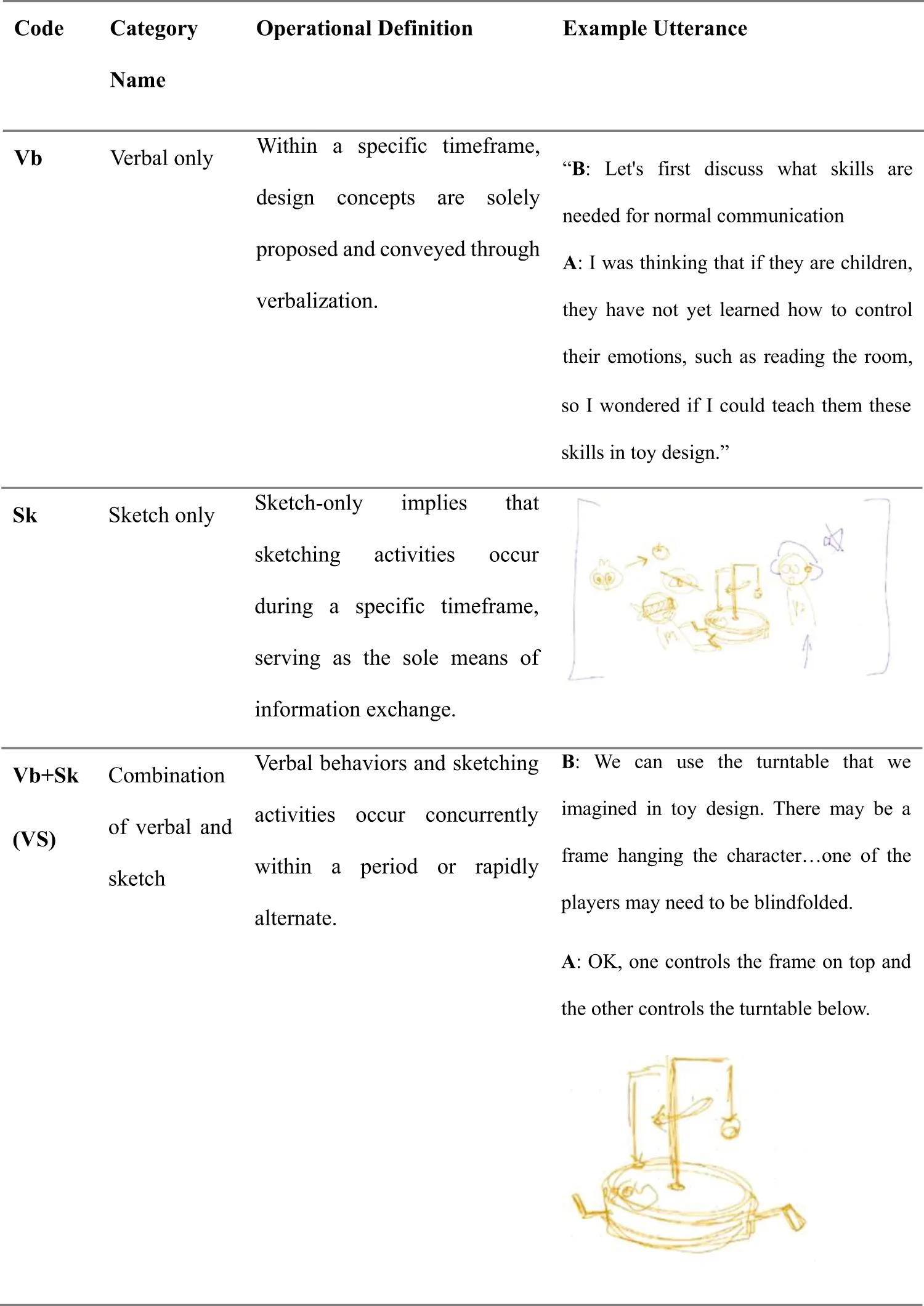
Coding scheme 2-Interaction behaviour

#### Preprocessing

The raw neural signals underwent a multi-step preprocessing routine to extract clean hemodynamic responses suitable for hyperscanning analysis. First, signals were bandpass filtered (0.01–0.10 Hz) with a third-order Butterworth filter to remove low-frequency drift and physiological noise. The filtering was implemented using the gsignal package in R. Following recent best practices for fNIRS hyperscanning (Jiang et al. 2015; Yang et al. 2020), we narrowed the bandpass to 0.01–0.1 Hz to exclude respiratory artefacts (~ 0.2–0.3 Hz) while preserving the canonical hemodynamic response band; this conservative upper bound also minimises residual cardiac interference and slow Mayer-wave contamination.

This cleaned signal was then segmented using a sliding-window approach with a 20-second window and a 1-second step. Next, a variance threshold of 4 SD was applied to identify and exclude windows contaminated by motion artefacts or poor optode-to-skin contact. After outlier removal, to ensure equal statistical weighting across conditions and dyads, 15 windows were randomly selected from the remaining pool for each condition-behaviour combination. Crucially, this random selection was conducted automatically based on behavioural coding timestamps, blind to the corresponding neurophysiological (IBS) data, to avoid selection bias. Following a final visual inspection to ensure data quality, the cleaned signals were averaged by hemisphere for each participant. This process yielded four final data streams representing the left and right frontal activity for each subject: Subject 1 Left Frontal (S1LF), Subject 1 Right Frontal (S1RF), Subject 2 Left Frontal (S2LF), and Subject 2 Right Frontal (S2RF).

#### Inter-brain synchrony (IBS) measurement

To quantify inter-brain synchrony (IBS) between dyadic pairs, we conducted two levels of analysis (group-level and ROI-level) to capture both the general nature of social connections and the spatial specifics of where neural coordination occurs. In this study, oxygenated hemoglobin (OxyHb) was selected as the primary signal of interest for calculating inter-brain synchrony (IBS). OxyHb is widely recognised as the most sensitive indicator of task-related changes in regional cerebral blood flow and is the standard metric in fNIRS hyperscanning literature (e.g., Cui et al. 2012; Jiang et al. 2012).

Phase Locking Value (PLV) was selected as the primary IBS metric. PLV provides a model-free measure of phase synchrony computed directly from the bandpassed signal via the Hilbert Transform, without requiring a secondary frequency-band selection parameter. This avoids the window-length-dependent spectral resolution constraints inherent in wavelet-based methods: because the time-frequency uncertainty principle ties the lowest resolvable frequency to the observation window length, a 20-second window cannot resolve frequencies below ~ 0.05 Hz with wavelet coherence, whereas PLV operates on the full bandpassed signal and is insensitive to this constraint (Lachaux et al. 1999). PLV quantifies the temporal consistency of instantaneous phase differences between the two filtered hemodynamic streams, with statistical significance assessed against 50 phase-surrogate samples. PLV values range from 0 (no phase locking) to 1 (perfect phase synchrony).

To ensure the methodological robustness of our findings and mitigate the risk of algorithm-specific artefacts, the IBS patterns observed via PLV were triangulated using three complementary metrics: Wavelet Transform Coherence (WTC), Mutual Information (MI), and Granger causality. WTC provides a localised measure of coherence in both the time and frequency domains, making it sensitive to transient episodes of neural coupling. To maximise frequency resolution for the supplementary WTC analysis, we computed wavelet coherence on the full filtered recording for each dyad and subsequently extracted per-window coherence values. This compute-first architecture decouples the WTC frequency band from the 20-second observation window, allowing analysis down to 0.01 Hz. For consistency with the bandpass, WTC was summarised within the 0.01–0.10 Hz frequency band. In parallel, MI was employed (configured with 10 bins) to capture non-linear statistical dependencies, offering an information-theoretic perspective on the dyads’ cognitive alignment, and Granger causality was computed to provide a directional (predictive) benchmark. This multi-method validation confirms that the observed synchrony is a stable, underlying phenomenon.

## Results

### Quantitative protocol analysis

Table 3 displays the descriptive statistics for the coded collaborative episodes, totaling 640 segments across both face-to-face and Zoom-based online modes. A preliminary analysis shows that co-exploration dominates the solution space more than the problem space in both contexts. While verbal strategies were very common in the in-person setting, the online environment showed a notable rise in hybrid interactional strategies, especially during solution generation, where VS made up 19.38% of observations. Regarding time allocation, hybrid interactions in the problem space within face-to-face sessions are the most time-consuming, averaging 61.70 s.

**Table 3:** Descriptive statistics

Collaboration modality	Design space focus	Interaction behaviour	Frequen-cy (*N*)	Proportion (%)	Mean time (s)	Median time (s)	SD Time (s)
Face-to-Face (F2F)	PS	Vb	91	14.22	49.98	43.00	33.51
		VS	20	3.13	61.70	60.00	43.48
		Sk	5	0.78	45.00	25.00	34.68
	SS	Vb	92	14.38	48.11	34.00	45.53
		VS	76	11.88	55.38	44.00	38.52
		Sk	48	7.50	60.23	42.50	41.80
Online via Zoom (OvZ)	PS	Vb	57	8.90	34.74	29.00	18.85
		VS	30	4.69	53.63	44.00	30.67
		Sk	2	0.31	47.00	47.00	18.38
	SS	Vb	49	7.66	38.07	36.50	19.95
		VS	124	19.38	48.43	40.00	27.69
		Sk	46	7.19	59.24	45.00	49.03

Proportion is calculated relative to the total number of observations (N = 640).

To assess the statistical significance of these behavioural shifts, two three-way repeated-measures ANOVAs (Modality ~ Design Space ~ Behaviour) were conducted, focusing on time-spent (Table 4) and frequency(Table 5)*.* The analyses revealed consistent patterns across both metrics. First, no significant main effects of collaborative modality were found for either time-spent or frequency. By contrast, highly significant main effects were observed for both “design space focus” (Time: *p* <. 001; Frequency: *p* <. 001) and “interactional strategy” (Time: *p* =.002; Frequency: *p* <.001), underscoring that the dyads’ cognitive and behavioural allocations were far from uniform.

Critically, the behavioural differences are illuminated by the significant interaction effects. A robust “Modality × Behaviour” interaction was identified for both time spent (*F* = 9.50, *p* =.001) and frequency (*F* = 16.34, *p* <.001). This demonstrates that while the total collaborative effort remained constant across environments, the specific interactional strategies dyads used to externalise their thoughts were strongly dictated by the environment. Furthermore, a significant “Design Space × Behaviour” interaction was also observed. This revealed that students adopt distinct externalisation strategies depending on whether they are framing the problem or actively generating solutions. The three-way interaction was not statistically significant in either model.

**Table 4:** Three-way repeated-measures ANOVA (Time spent)

Source	Num df	Den df	F	*p*
*Main effects*				
Modality	1	10	2.60	0.138
Design space	1	10	44.18	< 0.001***
Behaviour	2	20	9.09	0.002**
*Interactions*				
Modality × design space	1	10	2.09	0.169
Modality × behaviour	2	20	9.50	0.001**
Design space × behaviour	2	20	17.69	< 0.001***
3-way interaction	2	20	0.73	0.495

Significance level indicated by bold and asterisk (***p <.001, **p <.01, *p <.05).

**Table 5:** Three-way repeated-measures ANOVA (Frequency)

Source of variation	df	F	*p*
*Main effects*			
Modality	1	0.70	0.422
Design space	1	24.80	**< 0.001** ***
Behaviour	2	17.37	**< 0.001** ***
*Interactions*			
Modality × design space	1	1.52	0.246
Modality × behaviour	2	16.34	**< 0.001** ***
Design space × behaviour	2	25.09	**< 0.001** ***
3-way interaction	2	1.71	0.207

Significance level indicated by bold and asterisk (***p <.001, **p <.01, *p <.05).

**Fig. 5:**
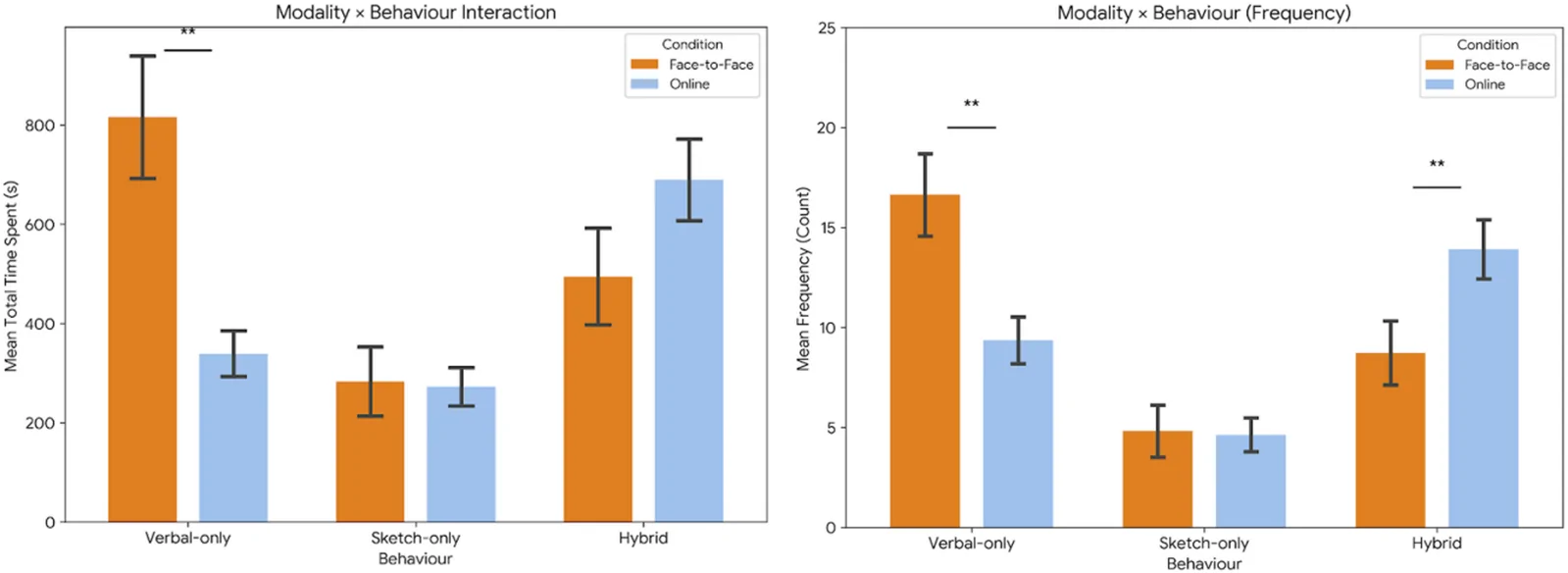
Modality by behaviour on time-spent and frequency. Error bars represent the standard error of the mean (SEM)

To clarify the specific factors driving the Modality × Behaviour interaction, post-hoc paired-samples t-tests were performed, as shown in Fig. 5. Results indicated that Face-to-Face (F2F) collaboration was mainly characterised by Verbal-only interactions. Participants spent significantly more time (*p* =.003, Cohen’s *d* = 1.15) and engaged in more frequent verbal exchanges (*p* =.002) in the F2F setting compared to the video-mediated environment. Conversely, switching to online settings led to a compensatory shift toward a hybrid communication strategy. Students exhibited a significantly higher frequency of combined behaviours when collaborating online (*p* =.008), but the difference in total time spent on hybrid behaviours between modalities was not statistically significant (*p* =.135). This suggests that the average duration of each hybrid interaction must be considerably shorter. Instead of participating in long, continuous blocks of hybrid behaviour (talking while sketching together), online pairs tend to engage in fragmented, “start-and-stop” interactions.

**Fig. 6:**
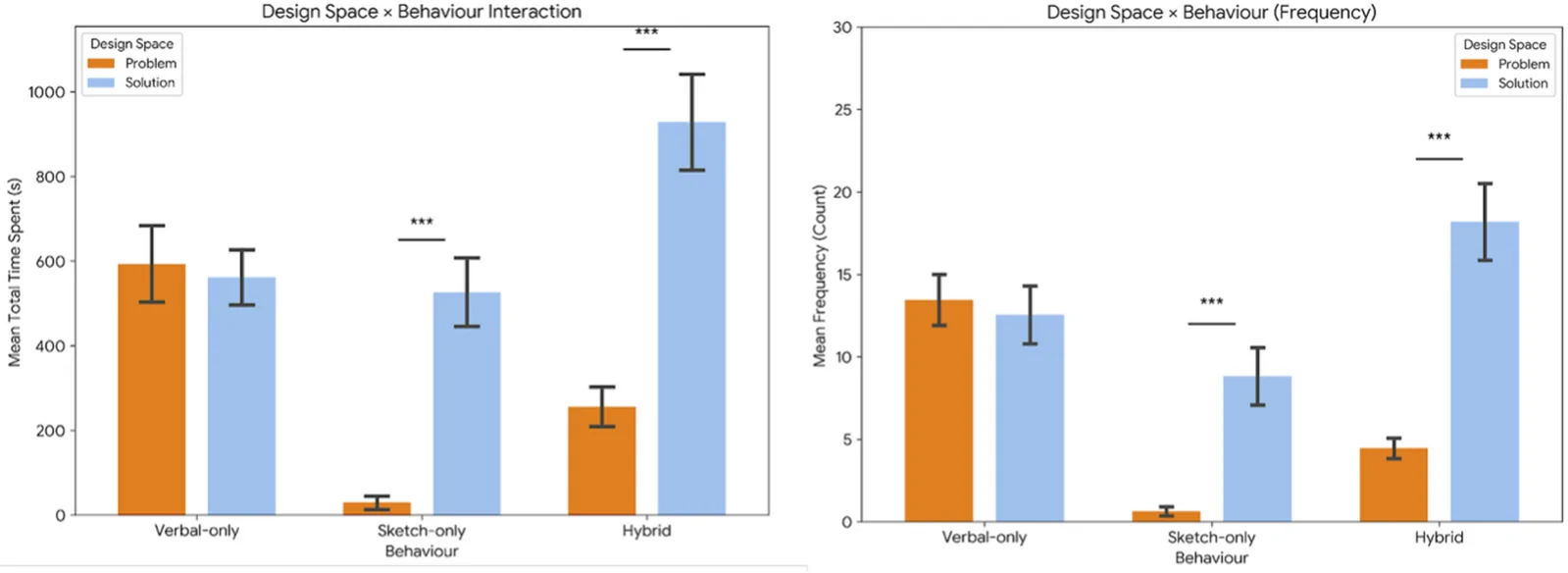
Design Space by Behaviour on time-spent and frequency. Error bars represent the standard error of the mean (SEM)

Further post hoc comparisons were used to decompose the Design Space × Behaviour interaction (Fig. 6), revealing a stark shift in externalisation strategies depending on the cognitive focus (problem framing and solution generation). Verbal-only communication served as a stable, foundational baseline, with no significant differences in either time (*p* =.687) or frequency (*p* =.591) between problem framing and solution generation. However, the solution generation phase required a substantial increase in sketching. Both sketch-only behaviours (Time: *p* <.001; Frequency: *p* <.001) and mixed behaviours (Time: *p* <.001; Frequency: *p* <.001) elevated significantly during solution space searching. The exceptionally large effect sizes (*d* > 1.8) underscore that active solution generation inherently demands external visual representations, regardless of whether the collaboration occurs physically or digitally.

### IBS results

To evaluate the effects of collaborative modality, interaction behaviours, and region of interest (ROI) on inter-brain synchrony (IBS), a three-way mixed-effects ANOVA (IBS ~ ROI_Pair × Behaviour × Condition) was conducted on the primary Phase Locking Value (PLV) metric, yielding 1,286 estimates across 11 dyads (see Table 7). The descriptive statistics can be found in Appendix A. The model yielded significant main effects for both Behaviour (*F* = 4.87, *p* =.008) and Condition (*F* = 28.95, *p* <.001). The two-way interaction between Behaviour and Condition was marginal and did not reach the conventional significance threshold (*F* = 2.88, *p* =.057), although it was highly significant in the supplementary MI (*F* = 29.46, *p* <.001) and WTC (*F* = 11.70, *p* <.001) models, suggesting that the modality dependence of behavioural coupling is most detectable with information-theoretic and wavelet metrics. Notably, the main effect of Region of Interest (ROI Pair) was non-significant (*F* = 1.72, *p* =.190), and there were no significant two-way or three-way interactions involving the ROI variable (all *p* >.23) Table 6.

**Table 6:** Three-way ANOVA: Mixed-effects model: IBS (PLV) ~ ROI_Pair × Behaviour × Condition + (1|Pair)

Effect	F	*p*
ROI_pair	1.72	0.190
Behaviour	4.87	**0.008**
Condition	28.95	**< 0.001**
ROI_pair: behaviour	3.11	**0.045**
ROI_pair: condition	0.76	0.383
Behaviour: condition	2.88	0.057
ROI_pair: behaviour: condition	1.44	0.237

Significant effects (p <.05) are shown in bold. IBS quantified by Phase Locking Value (PLV), the primary metric.

Following this overall model, planned post-hoc contrasts on the PLV data (see Table 7) revealed a significant main effect of collaborative modality, with face-to-face (F2F) interaction exhibiting a higher overall baseline of inter-brain synchrony (IBS) compared to the online condition (est = 0.073, *t* = 5.38, *p*.fdr < 0.001). This modality effect was strongly conditional on the specific interactional strategy. Decomposing the effect by behaviour, sketching was associated with the most robust neural alignment advantage for F2F (est = 0.115, *t* = 4.93, *p*.fdr < 0.001), and hybrid interaction showed a comparable F2F advantage (est = 0.067, *t* = 2.89, *p*.fdr = 0.006). In contrast, the modality effect for verbal-only interaction did not reach significance (est = 0.036, *t* = 1.53, *p*.fdr = 0.126), indicating that verbal coupling was largely preserved across physical and digital settings. Within the F2F condition, sketching yielded significantly higher IBS than verbal-only interaction (est = −0.077 for Verbal - Sketch, *t* = −3.20, *p*.fdr = 0.015), corroborating that sketching is the behaviour most strongly associated with frontal coupling in co-located design collaboration. At the ROI × Behaviour level, the F2F sketching advantage was significant in both homologous hemisphere pairs (L-L: est = 0.109, *t* = 3.29, *p*.fdr = 0.002; R-R: est = 0.122, *t* = 3.70, *p*.fdr = 0.001). Thus, the PLV primary metric paints a more parsimonious picture: the F2F advantage holds overall and is driven principally by sketching (and combined) behaviours, while verbal-only coupling is comparably weak in both modalities. The behavioural shift toward fragmented hybrid interaction online is therefore best characterised as a relative attenuation of sketching-associated coupling rather than a compensatory surge in verbal coupling.

However, although our analysis did not reveal significant differences between the Regions of Interest (ROIs), this finding must be interpreted with extreme caution. Because our 8-channel system required averaging signals across the hemisphere (collapsing distinct regions such as the DLPFC, VLPFC, and mPFC into broad left/right streams), this spatial smoothing severely limits spatial resolution. Therefore, the absence of an observed ROI effect is highly likely a consequence of this methodological averaging rather than true neurobiological uniformity across the prefrontal cortex.

**Table 7:** Summary of significant post-hoc comparisons (PLV, primary metric)

Comparison level	Condition focus	Contrast (difference)	Estimate	SE	t-value	*p* (FDR)
Main effects of modality	Overall	F2F - online	0.073	0.014	5.38	**< 0.001**
Modality within behaviour	Sk	F2F - online	0.115	0.023	4.93	**< 0.001**
	Vb	F2F - online	0.036	0.024	1.53	0.126
	VS	F2F - online	0.067	0.023	2.89	**0.006**
Modality within ROI×behaviour	L-L (Sk)	F2F - online	0.109	0.033	3.29	**0.002**
	R-R (Sk)	F2F - online	0.122	0.033	3.70	**0.001**
Behaviour within modality	F2F	Vb- Sk	−0.077	0.024	−3.20	**0.015**

Significant effects (p <.05) are shown in bold. *F2F* - face-to-face, *Sk* sketch-only, *Vb* verbal-only, *VS* verbalisation and sketching, *L-L* left-to-left hemisphere, *R-R* -right-to-right hemisphere, *Cross* - cross-hemisphere.

To validate the stability and robustness of our findings, Table 8 presents a re-computation of the core headline contrasts using the full N = 11 sample. Unlike the primary bootstrap-aggregated approach (V2 architecture) reported previously, the estimates in Table 8 are derived from an independently run linear mixed-effects model fitted directly to the per-window Phase Locking Value (PLV) estimates (V1 architecture). As anticipated, while the magnitudes of the estimates differ between the two analytical architectures, the overall direction of the effects remains strictly consistent, thereby corroborating the reliability of the observed neuro-behavioural shifts. Notably, specific Region-of-Interest (ROI) by Behaviour contrasts are intentionally omitted from this re-computation; the main effect of ROI was non-significant in this omnibus model, and the inherent spatial smoothing from hemispheric averaging precludes reliable sub-regional inference at a pilot sample size of N = 11. Finally, to rigorously account for multiple testing, the right-most column reports *p*-values adjusted via the Benjamini-Hochberg False Discovery Rate (FDR) procedure, treating these specific a priori contrasts as a single hypothesis family.

**Table 8:** N = 11 full-sample recomputed post-hoc contrasts (PLV, primary metric; V1 per-window mixed-effects model)

Comparison level	Condition focus	Contrast (difference)	Estimate	SE	t-value	*p* (raw)	*p* (BH-FDR)
Main effects of modality	Overall	F2F − online	0.058	0.019	3.29	**0.004**	**0.020**
Modality within behaviour	Sk	F2F − online	0.074	0.029	2.51	**0.028**	0.067
Modality within behaviour	Vb	F2F − online	0.049	0.029	1.55	0.138	0.276
Modality within behaviour	VS	F2F − online	0.052	0.029	1.62	0.118	0.276
Behaviour within modality	F2F	Vb − Sk	−0.065	0.034	−2.01	0.060	0.135
Behaviour within modality	F2F	VS − Sk	−0.069	0.034	−2.10	0.050	0.135

Significant effects (p <.05) are shown in bold.

**Fig. 7:**
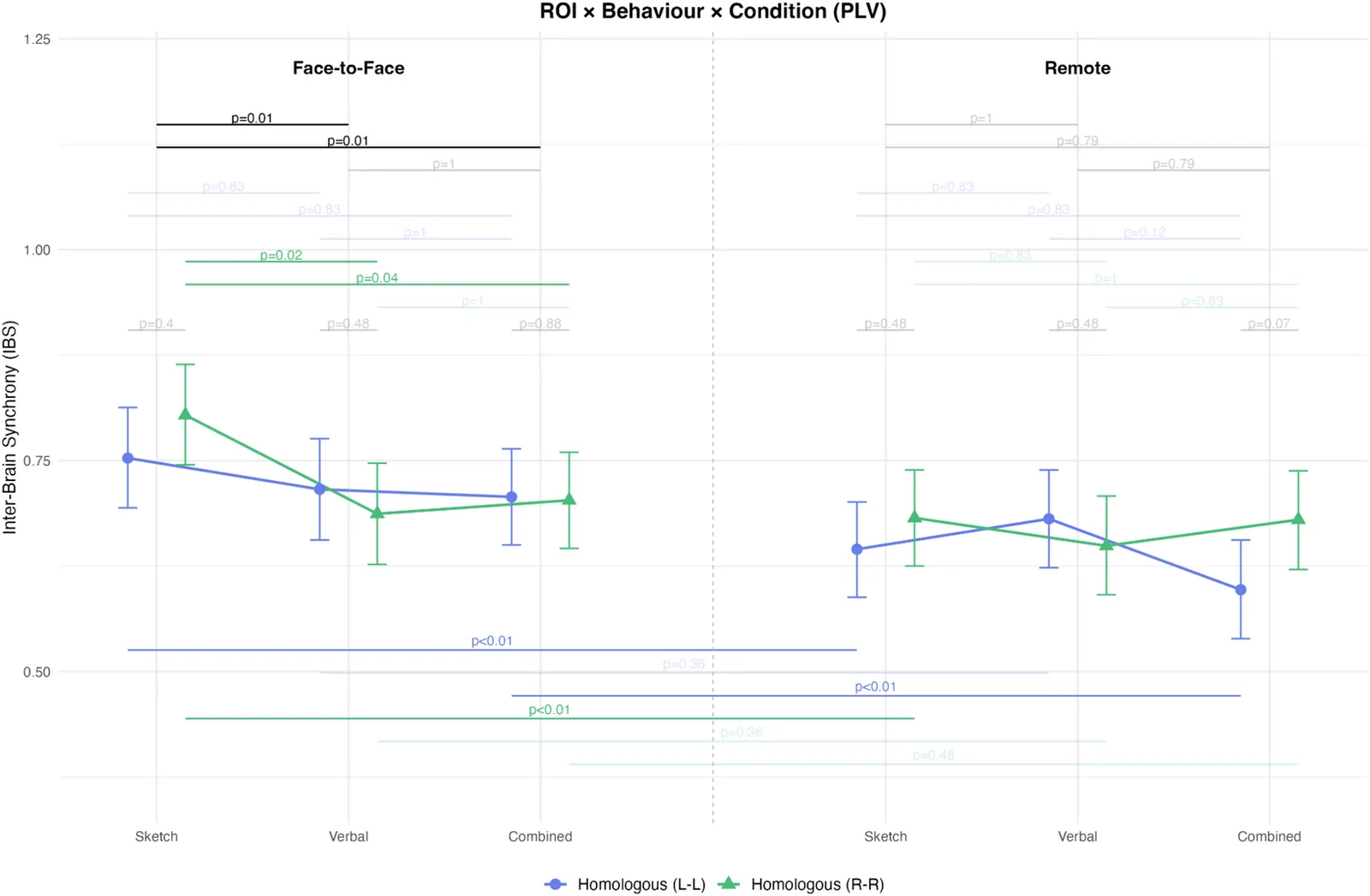
ROI_Pair × Behaviour × Condition (PLV)

**Fig. 8:**
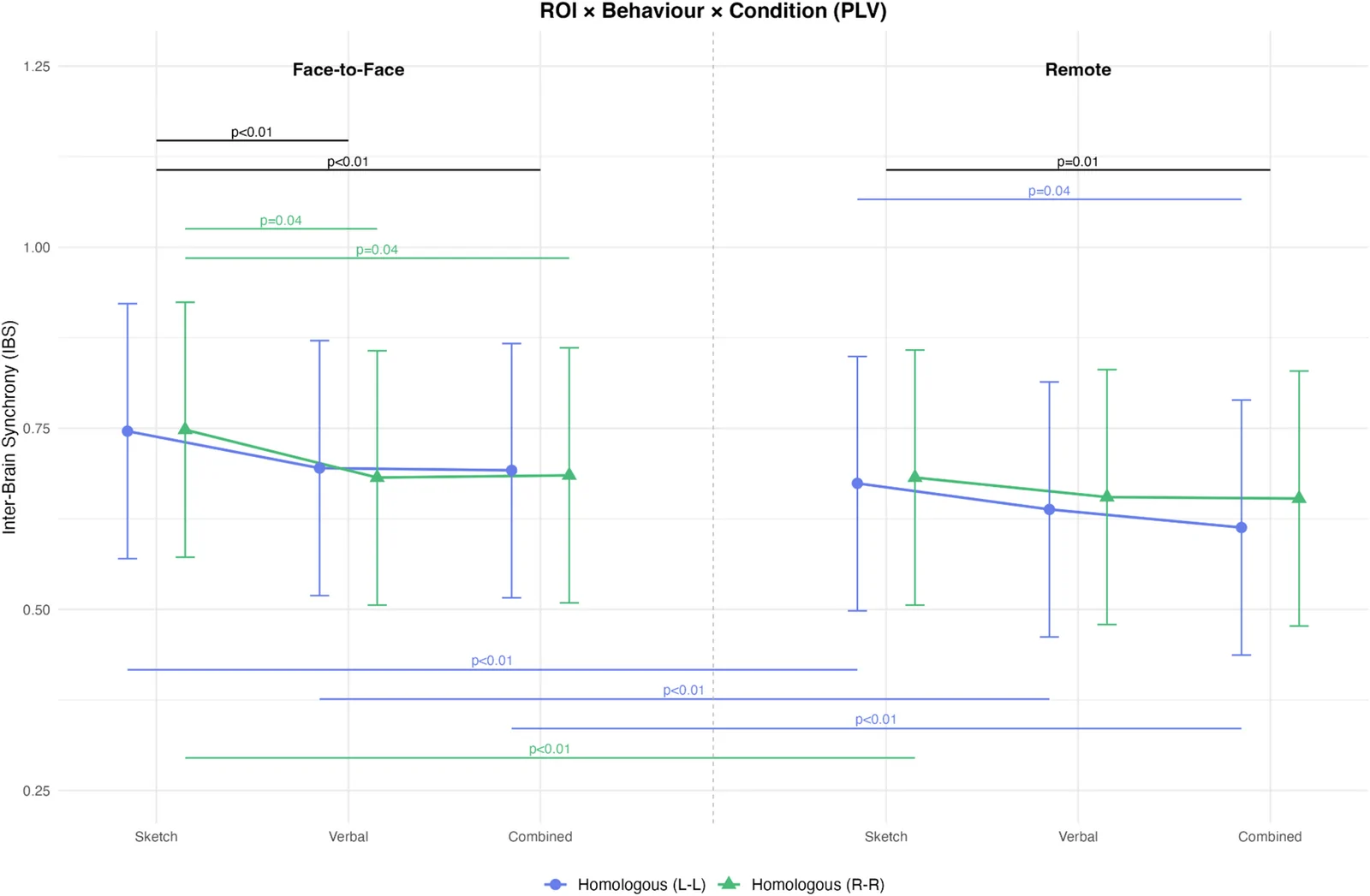
ROI × Behaviour × Condition (PLV), *N* = 11 recomputation with non-significant pairwise comparisons correctly hidden (cf. Figure 7, which displays all comparisons including non-significant ones due to a leftover debug setting -- see accompanying comment). Homologous ROI pairs only (Left-Left, Right-Right); estimates from the V1 per-window architecture (cf. Table 8 notes)

Beyond the aggregate main effects reported above, the ROI-resolved post hoc contrasts underlying Fig. 8 reveal a behaviour-dependent hemispheric asymmetry that has not been previously discussed. The face-to-face advantage in inter-brain synchrony was bilaterally robust for sketching, reaching significance in both the left-left (est = 0.073, p.fdr = 0.002) and right-right (est = 0.066, p.fdr = 0.004) homologous channel pairs. By contrast, the face-to-face advantage for verbal-only (est = 0.057, p.fdr = 0.010) and combined verbal-plus-sketching (est = 0.079, p.fdr = 0.001) behaviours reached significance only in the left-left pairing, with the corresponding right-right contrasts falling short of significance.

This pattern broadly consistent with the established distinction between left-hemisphere language networks, which favour focal, predominantly interhemispheric interactions, and right-hemisphere networks supporting integrative, more bilaterally distributed processing (Gotts et al. 2013). Sketching recruits distributed bilateral fronto-parietal circuitry supporting visuospatial planning and motor execution (Raimo et al. 2021), which may explain why its associated inter-brain coupling was detectable across both hemispheres, whereas the more language-dependent verbal and combined behaviours showed a coupling advantage concentrated in the left, language-dominant pairing. We present this pattern as a descriptive observation from planned contrasts rather than a pre-registered hypothesis, and it should be interpreted with the same caution warranted by our 8-channel hemispheric averaging (discussed above). Nonetheless, it offers a physiologically plausible account of why the two homologous ROI pairs are not interchangeable once behaviour is taken into account, and it motivates finer-grained regional analysis in future work with higher-density optode arrays.

To confirm that the observed synchrony patterns were robust, parallel statistical analyses were conducted using the three supplementary metrics: Wavelet Transform Coherence (WTC), Mutual Information (MI), and Granger causality (see Supplementary Fig. 1,2,3). The MI model yielded convergent results, replicating the significant main effects of Behaviour and Condition and the Behaviour × Condition interaction, with the same F2F > Remote direction observed in the primary PLV analysis (MI main effect: est = 0.171, *t* = 12.14, *p*.fdr < 0.001; Sketch: est = 0.323, *p*.fdr < 0.001). Notably, the supplementary WTC analysis in the narrow 0.01–0.1 Hz band produced a directionally inconsistent main effect (est = −0.038, *p*.fdr < 0.001; i.e., Remote > F2F), a metric-specific reversal that we attribute to the sensitivity of wavelet coherence to residual low-frequency autocorrelation when the analysis band is restricted to the hemodynamic floor. Reassuringly, when WTC was computed under the original, broader 0.01–0.9 Hz bandpass (Sensitivity Analysis C below), the F2F > Remote direction was preserved (est = 0.121, *p*.fdr < 0.001), confirming that the sign reversal is specific to the narrow-band wavelet implementation rather than to the underlying neural data. Full statistical reporting for the WTC, MI, and Granger models is available in the supplementary materials (Appendix B). This cross-methodological triangulation indicates that the core modality effect is stable under phase-based (PLV) and information-theoretic (MI) metrics, while the wavelet metric should be interpreted with caution in the narrow band.

### Sensitivity analysis

To verify that the primary finding (F2F > Online inter-brain synchrony, driven principally by sketching) was not an artefact of a single analytic configuration, we re-ran the full PLV three-way model across five sensitivity variations. The main modality effect (F2F > Remote) remained significant and directionally consistent across all five configurations, and the sketch-specific F2F advantage was preserved in every case:

*Sensitivity A* (30-second window): Main effect est = 0.080, *p*.fdr < 0.001; Sketch *p*.fdr = 0.005.

*Sensitivity B* (broader bandpass 0.01–0.2 Hz): Main effect est = 0.104, *p*.fdr < 0.001; all three behavioural conditions significant.

*Sensitivity C* (original 0.01–0.9 Hz bandpass, legacy architecture): Main effect est = 0.121, *p*.fdr < 0.001; all three behavioural conditions significant.

*Sensitivity D* (deoxyhemoglobin, HHb): Main effect est = 0.042, *p*.fdr = 0.003; Sketch *p*.fdr < 0.001; Verbal null (*p*.fdr = 0.964), mirroring the OxyHb PLV pattern.

*Sensitivity E* (alternative random seed, seed = 42): Main effect est = 0.060, *p*.fdr < 0.001; Sketch *p*.fdr < 0.001.

*Sensitivity F* (structured 12-condition matrix, full N = 11 sample): across the twelve band-pass × window combinations described in Methods, the modality × sketching interaction maintained the same direction (face-to-face greater than remote) in every configuration when Phase Locking Value was used as the synchrony metric, with full-sample p-values ranging from 3.0 × 10⁻⁶ (narrowest band-pass) to 2.95 × 10⁻¹² (widest band-pass). Mean cluster-bootstrapped effect sizes ranged from 0.058 to 0.078, depending on the specific band-pass configuration.

For wavelet coherence, whose effective frequency band varies across the twelve configurations, effect sizes were an order of magnitude smaller than those for Phase Locking Value (0.001 to 0.007), and the narrowest wavelet band tested (0.10–0.167 Hz) produced a small sign reversal (estimate = − 0.001, p =.98). This pattern is consistent with the known sensitivity of wavelet-coherence estimates to frequency-band selection at low signal-to-noise ratio, and directly corroborates the concern that motivated our adoption of Phase Locking Value as the primary synchrony metric (see Methods 3.4).

A cluster-bootstrap procedure (1,000 iterations per condition, resampled at the dyad level) was used to assess the stability of these effects across the full twelve-condition matrix. The proportion of bootstrap iterations that reproduced the sign of the full-sample effect ranged from 0% to 17% across the twenty-four cells of the matrix. We report this range candidly: it reflects the expected limits of cross-dyad generalisation at N = 11 rather than a weakness specific to any single analytic configuration, and it should be read alongside, not instead of, the full-sample significance tests.

In every configuration, the estimate was positive (F2F > Online) and statistically significant after FDR correction, and the sketching contrast consistently had the largest magnitude. These results establish that the core finding is robust across window lengths, bandpass settings, haemoglobin species, and resampling seeds.

## Discussion

Two methodological clarifications are worth noting before interpreting the discussion below. First, the estimates reported in Table 8 (and the accompanying Sensitivity F analysis) are smaller in magnitude than those in Table 7, despite being derived from the same eleven-dyad sample; this reflects a difference between the per-window mixed-effects (‘V1’) and bootstrap-aggregated (‘V2’) analytic architectures rather than any change in the underlying data or sample composition. Both estimates point in the same direction and reach the same qualitative conclusion.

The primary objective of this exploratory pilot study (N = 11 dyads) was to delineate how the mode of collaboration (Face-to-Face versus Online via Zoom) modulates both observable interactional behaviours and the underlying inter-brain synchrony (IBS) among design students. Given the sample size constraints inherent to fNIRS hyperscanning, this investigation was designed to identify preliminary neuro-behavioural associations rather than establish definitive causal mechanisms. Our initial findings suggest that Face-to-Face (F2F) collaboration establishes a significantly higher overall baseline of inter-brain synchrony (IBS) than digital environments, corroborating a consensus within recent hyperscanning literature (Hu et al. 2025; Sato and Sato 2025). However, the transition from a shared physical environment to a digital interface appears to coincide with a notable inversion of the strategies associated with neural coupling, highlighting the potential cognitive impacts of interactional friction in remote design education.

### The attenuation of sketching-associated neural coupling between face-to-face and online settings

Our multimodal analysis revealed a notable contrast between observable collaborative behaviour and underlying neural synchrony. Protocol analysis showed that Face-to-Face (F2F) environments encouraged significantly more frequent and longer verbal-only interaction episodes. In contrast, the transition to an online environment (via Zoom) resulted in a significant shift towards hybrid work, with increased sketching. However, subsequent analysis of inter-brain synchrony (IBS) within the prefrontal cortex revealed a partially inverted pattern. In the F2F setting, although sketch-only behaviours accounted for the smallest proportion of overall time, they generated the highest level of IBS, emerging as the behaviour most strongly associated with neural coupling (F2F Sketch > F2F Verbal, est = 0.077, *p*.fdr = 0.015). Conversely, when collaborating online, this sketching-associated neural synchrony diminished significantly (Sketch F2F > Remote, est = 0.115, *p*.fdr < 0.001). Verbal-only coupling, by contrast, did not differ significantly between modalities (est = 0.036, *p*.fdr = 0.126), indicating that verbal communication sustains a comparable, if modest, level of frontal coupling in both settings. Taken together, these findings suggest that physical co-presence strongly facilitates sketching-associated cognitive alignment, whereas remote dyads lose this sketching-coupling advantage without a corresponding compensatory surge in verbal coupling under the PLV metric.

This notable neuro-behavioural inversion can be understood through the lens of embodied cognition and the disembodiment of the digital trace. In physical settings, sketching is inherently embodied. As Tang (1991) established in foundational CSCW research, collaborative drawing is less about the final artefact and more about mediating interaction through shared gaze, hand gestures, and body language. When a student sketches physically, their partner’s neural network synchronises not only with the drawing but also with the shared intentionality of the movement. It is a highly concentrated, visually rich alignment approach that requires minimal time to produce massive executive synchronisation during complex design problem-solving (Goel 1995).

Conversely, in a digital environment, the act of drawing is decoupled from the actor’s physical body. Although collaborators can see digital strokes emerge, they are deprived of the preceding deictic cues (e.g., watching a partner’s eyes dart to a specific corner before their hand moves). Recent neuroimaging studies have found that the brain processes live, physical partners entirely differently from video-streamed interactions. This disparity often results in reduced neural coherence between participants during collaborative problem-solving in digital settings (Balters et al. 2023). Because the digital sketch lacks embodied context, it usually leaves dyads visually active yet neutrally decoupled.

To compensate for the weakened visuospatial channel, online dyads were observed to shift towards more explicit verbalisation. Collaborators need to establish “common ground” to achieve mutual understanding (Clark and Brennan 1991). In the absence of reliable physical cues, online students must actively and explicitly narrate their design intent to maintain alignment. Our behavioural data reveal a significant increase in bursts of short, fragmented hybrid interactions (sketching while talking). However, under the primary PLV metric, this compensatory verbalisation did not translate into a statistically significant elevation of verbal-only IBS in the remote condition (verbal F2F - Online: est = 0.036, *p*.fdr = 0.126); the supplementary MI metric did detect a significant verbal F2F > Online effect (est = 0.097, *p*.fdr < 0.001), leaving the “compensatory verbal coupling” account as a partially supported, metric-dependent interpretation rather than a confirmed finding. This high-intensity verbal narration plausibly demands substantial cognitive effort, which may contribute to the elevated verbal IBS observed in the video-mediated condition under information-theoretic metrics.

### The vulnerability in digital collaborative solution generation

The protocol analysis revealed a pronounced asymmetry in how design dyads distributed their collaborative efforts, with the dataset heavily skewed towards co-exploring the Solution Space (~ 68% of all episodes) rather than the Problem Space. Critically, the transition into the idea-generative phase was accompanied by a marked increase in sketching activity. The behavioural data demonstrate that both sketch-only and hybrid (verbal + sketch) episodes occurred far more frequently and lasted significantly longer during solution generation than during problem framing. As Schön’s theory (1983) articulated, sketching serves multiple cognitive functions and is not merely a recording tool but an active, driving mechanism for thought. Suwa et al. (1998) also articulated that sketches act as a “physical setting in which design thoughts are constructed on the fly”. A robust body of literature confirms this generative power of sketching: sketching empowers designers to explore a broader “idea distance”, resulting in more creative final concepts (Bao et al. 2016), and significantly outperforms purely internal mental imagery for novice idea generation (Tedjosaputro MA 2018).

Our Face-to-Face (F2F) neurophysiological data strongly supports the importance of visual externalization for mutual understanding. The fact that interactions using only sketches produced the highest IBS in physical environments (F2F Sketch > F2F Verbal, *p*.fdr = 0.015) highlights how shared, embodied sketching closely links the prefrontal executive functions of design students.

However, the attenuation of sketching-associated neural synchrony in the digital environment reveals a critical vulnerability in online design collaboration. The behaviour most empirically vital for solution generation (sketching) is the very behaviour whose inter-brain coupling is most diminished across digital interfaces (Sketch F2F - Online: est = 0.115, *p*.fdr < 0.001). Consequently, during the complex, generative phase of the design learning process, online students experience a relative reduction in sketching-mediated frontal coupling. While they may be drawing on the same digital canvas, the prefrontal networks supporting that shared sketching activity are less coupled than in co-located settings, which may heighten the risk of misaligned mental models, localised fixation, and collaborative breakdowns. We emphasise that these downstream outcomes remain hypothesised risks requiring outcome-based follow-up rather than demonstrated consequences of the observed neural dynamics.

While embodied cognition provides a useful framework for understanding the disruption of neural coupling caused by sketching in digital spaces, it is important to recognize that this is not the only explanation. First, the decline in inter-brain synchrony (IBS) might mainly result from the cognitive load of video conferencing (such as “Zoom fatigue,” managing self-view, and processing asynchronous audiovisual cues), which can divert mental resources from collaborative alignment. Second, even though the shared iPad interface was used in both settings, its novelty and specific challenges may have affected participants more when physical presence and immediate troubleshooting were absent in remote conditions. Currently, the data are correlational and cannot definitively confirm the embodied cognition hypothesis over these alternative factors. Future research should aim to isolate these variables to pinpoint which aspects of remote work reduce shared neural states.

## Limitation and future works

While this study provides a multimodal analysis of design collaboration across physical and digital environments, several limitations must be acknowledged to guide future research endeavours.

First, regarding the frequency band selection: while the 0.01–0.1 Hz bandpass removes respiratory artefacts (~ 0.2–0.3 Hz), residual overlap with Mayer waves (0.05–0.1 Hz) remains. However, Mayer waves are systemic blood pressure oscillations not expected to be phase-locked between dyad members, mitigating this concern for inter-brain analyses. The robustness of the core finding across five sensitivity configurations, including a broader 0.01–0.2 Hz bandpass that admits more of the Mayer-wave band, further indicates that this residual overlap does not drive the observed modality effect.

A limitation concerns cross-dyad generalisation given the small sample size. The cluster-bootstrap analysis (Sensitivity F) showed only 0–17% of resampling iterations reproduced the full-sample effect’s sign across twelve configurations. This doesn’t disprove the finding; the full-sample estimates are statistically significant and consistent, but the effect’s precise magnitude is provisional until replicated in a larger sample. We suggest at least twenty dyads in future work, which could increase bootstrap sign-reproduction to a more acceptable 3030303030303030% or higher.

Second, it is critical to acknowledge that the pilot-scale sample size (N = 11 dyads) inherently limits the statistical power of our three-way interaction model (Modality × Interactional Behaviour × ROI). Given this limited power, the non-significant ROI effects in our data may be prone to Type II errors (false negatives). We cannot definitively conclude that IBS operates uniformly across the measured frontal regions; rather, the current sample size may lack the statistical sensitivity required to detect subtle regional variations during naturalistic collaboration. Furthermore, to avoid exacerbating this power limitation, the cognitive design space focus (Problem vs. Solution) identified in the behavioural coding was not incorporated into the fNIRS analysis. Future research utilising larger, adequately powered cohorts is essential to confidently resolve potential ROI differences and to investigate how neural synchrony fluctuates as designers temporally navigate between the problem and solution spaces.

Another critical limitation is that the neuroimaging scope of this pilot study was strictly constrained by the hardware configuration of the portable 88888888-channel fNIRS system, limiting data collection to the frontal regions (PFC). More critically, by not measuring posterior regions or the temporoparietal junction (TPJ), which are universally recognised as critical hubs for social interaction and mentalising, this study cannot capture the full spectrum of social neural synchrony. Instead, our findings must be narrowly interpreted as reflecting synchronised executive functioning and cognitive load. The PFC is primarily responsible for working memory, cognitive flexibility, and executive control required for generative design tasks. Thus, the inter-brain synchrony observed in this study represents the shared cognitive effort of the design process, rather than a holistic picture of social interaction. Future research utilising high-density, whole-head fNIRS montages is essential to map the complete socio-cognitive networks underlying remote collaboration.

This pilot study’s findings are limited to the specific software and hardware used. The digital environment was confined to a shared tablet-based whiteboard via Zoom, which offers features such as single-pointer interaction, inherent latency, and limited gaze cues. Therefore, the neuro-behavioural dynamics observed should not be generalised to all digital design settings. Platforms with different affordances, like multi-cursor desktop tools (e.g., Miro, Figma) or immersive spatial systems (e.g., Virtual Reality), may produce completely different inter-brain synchrony patterns.

Finally, this study did not correlate the observed IBS patterns or behavioural frequencies with the ultimate quality of the collaborative learning. Although high neural synchrony suggests deep cognitive alignment and shared intentionality, it does not guarantee a superior collaborative learning outcome. In the context of collaborative design, for instance, dyadic teams may exhibit high neural synchrony even while experiencing “design fixation”. Future studies must bridge this gap by employing validated expert evaluation rubrics to assess the final design outcomes.

## Conclusion

This preliminary study investigated the neuro-behavioural dynamics of collaborative design, specifically examining how the communication medium (physical Face-to-Face versus digital Online via Zoom) modulates interactional strategies and underlying inter-brain synchrony (IBS). Recognising the rapid digitalisation of design education, the primary objective was to bridge the gap between observable collaborative behaviours and the hidden cognitive coupling required for successful co-exploration. To achieve this, we employed a rigorous multimodal experimental paradigm, integrating quantitative protocol analysis (coding for “interactional behaviours” and “design space focus”) with fNIRS hyperscanning to capture executive neural synchronisation within the prefrontal cortex.

The findings revealed a potential neuro-behavioural pattern across two collaborative modalities, grounded in the primary Phase Locking Value (PLV) metric and corroborated by Wavelet Transform Coherence (WTC) and Mutual Information (MI). While Face-to-Face interactions were behaviourally dominated by verbal exchanges, sketching emerged as the behaviour most strongly associated with neural synchrony in co-located settings. Conversely, collaboration in digital environments shifts towards a fragmented, hybrid mode of interaction, characterised by simultaneous verbal description and sketching. Crucially, neurophysiological data indicate that, in online settings, sketching-associated inter-brain synchrony is significantlnated (est = 0.115, *p*.fdr < 0.001), and verbal-only coupling does not differ significantly between modalities, leaving online dyads without the sketching-mediated frontal coupling that characterises physical co-presence. By integrating these datasets, this study exposed a critical vulnerability in videoconferencing-based collaborative design: while the generative “solution space” relies heavily on sketching to drive the evolution of ideas, digital sketching in online environments is associated with reduced frontal coupling relative to face-to-face collaboration, which may heighten the hypothesised risk of misaligned mental models and collaborative breakdown.

## Supplementary Information

Below is the link to the electronic supplementary material.

Supplementary Material 1

## Data Availability

The datasets generated and/or analyzed during the current study are not publicly available due to privacy but are available from the corresponding author upon reasonable request.
