# Supplementary material for: Comparing design students’ inter-brain synchrony between face-to-face and online collaborations
Source: Brain Struct Funct. 2026 Sep 4;231(8):132. doi: 10.1007/s00429-026-03188-4 (PMC13545068; doi:10.1007/s00429-026-03188-4)
Supplement: Supplementary file 1 [file 429_2026_3188_MOESM1_ESM.docx]

**Appendix A**

**Supplementary Table 1a.** Statistics by Behaviour.

| Behaviour | N | Mean IBS | SD | SE | 95% CI |
| --- | --- | --- | --- | --- | --- |
| Sketch-only (Sk) | 800 | 0.576 | 0.264 | 0.009 | [0.557, 0.594] |
| Verbal-only (Vb) | 800 | 0.485 | 0.227 | 0.008 | [0.469, 0.500] |
| Verbal + Sketch (VS) | 832 | 0.485 | 0.228 | 0.008 | [0.469, 0.500] |

**Supplementary Table 1b.** Statistics by Modality.

| Modality | N | Mean IBS | SD | SE |
| --- | --- | --- | --- | --- |
| Face-to-Face (F2F) | 1180 | 0.555 | 0.257 | 0.007 |
| Online via Zoom (OvZ) | 1252 | 0.476 | 0.224 | 0.006 |

**Supplementary Table 1c.** Statistics by ROI Pair Type.

| ROI Pair | N | Mean IBS | SD | SE | 95% CI |
| --- | --- | --- | --- | --- | --- |
| **Homologous (Left-Left)** | 608 | 0.493 | 0.237 | 0.010 | [0.043, 1.000] |
| **Homologous (Right-Right)** | 608 | 0.521 | 0.245 | 0.010 | [0.018, 1.000] |
| Cross-hemisphere | 1216 | 0.522 | 0.246 | 0.007 | [0.016, 1.000] |

**Appendix B**

**Supplementary Table 2a.** Three-way ANOVA: Mixed-effects model: IBS (WTC) ~ ROI_Pair × Behaviour × Condition + (1|Pair).

| Effect | F | p |
| --- | --- | --- |
| ROI_Pair | 0.060 | .548 |
| Behaviour | 16.50 | **<.001** |
| Condition | 33.35 | **<.001** |
| ROI_Pair:Behaviour | 0.19 | .944 |
| ROI_Pair:Condition | 0.02 | .978 |
| Behaviour:Condition | 55.20 | **<.001** |
| ROI_Pair:Behaviour:Condition | 0.12 | .947 |

*Significant effects (p < 0.05) are shown in bold. IBS is quantified by Wavelet Transform Coherence (WTC), the primary metric.*

**Supplementary Table 2b.** Three-way ANOVA: Mixed-effects model: IBS (MI) ~ ROI_Pair × Behaviour × Condition + (1|Pair).

| Effect | F | p |
| --- | --- | --- |
| ROI_Pair | 7.11 | **<.001** |
| Behaviour | 71.11 | **<.001** |
| Condition | 201.87 | **<.001** |
| ROI_Pair:Behaviour | 0.13 | .970 |
| ROI_Pair:Condition | 0.90 | .406 |
| Behaviour:Condition | 38.94 | **<.001** |
| ROI_Pair:Behaviour:Condition | 0.15 | .963 |

*Significant effects (p < 0.05) are shown in bold. IBS is quantified by Mutual Information (MI), the primary metric.*

**Supplementary Table 2c.** Three-way ANOVA: Mixed-effects model: IBS (Granger) ~ ROI_Pair × Behaviour × Condition + (1|Pair).

| Effect | F | p |
| --- | --- | --- |
| ROI_Pair | 4.97 | **.002** |
| 2Behaviour | 17.76 | **<.001** |
| Condition | 38.24 | **<.001** |
| ROI_Pair:Behaviour | 1.21 | .300 |
| ROI_Pair:Condition | 2.37 | .068 |
| Behaviour:Condition | 2.25 | .105 |
| ROI_Pair:Behaviour:Condition | 0.37 | .895 |

*Significant effects (p < 0.05) are shown in bold. IBS is quantified by Granger, the primary metric.*
